# Plaster-of-Paris Bandage

**Published:** 1877-12-20

**Authors:** 


### PLASTER-OF-PARIS BANDAGE.

Dr. J. McLean, in a paper before the Southern Illinois Medical Association, remarks of the plaster-of paris bandage:

"In 1852 it began to assert its supremacy, and now stands at the head of the list.

The advantages of the immovable dressing may be summed up as follows: Perfect co-aptation to irregularities of the limb, and, as a result of this, little tendency to displacement of splint; less trouble to the surgeon; complete fixation of fragment; freedom of patient to go on crutches or otherwise; less irritation, less extravastion of blood, less swelling; no injurious pressure over bony prominences; less liability of excoriation; uniform compression giving muscular rest, diminishing spasm and removing one cause of displacement, preventing swelling, and lessening it where it exists. Simplicity of materials, and cheapness, and extensive range of application. The manner of application that we will notice consists in the roller and blanket splints, used separate or combined. Their preparation will be detailed as we notice the fractures treated.

Simple fractures of the tibia, or both tibia and fibula, low down, are treated in the following manner: Take four or five rollers of canton flannel, or common flannel, five yards long, and the usual width, into which rub freely dry plaster of paris, and roll loosely; have the limb extended, and the fragments of bone replaced. First apply a dry roller from the base of the toes to the knee, then dip the plastered roller in warm water for four or five minutes, squeeze out the superfluous water, and apply in the same manner and extent as the dry roller; four or five rollers thus applied are sufficient; extension should be maintained until the dressing becomes firm. Fractures of the bones high up should be treated as above described, except the dressing should be extended to the middle of the thigh.

In compound fractures a trap should be cut in the dressing before it becomes firm, large enougn to expose the entire wound, through which it can be inspected, dressed and discharges allowed to escape. To prevent a soaking up of any discharge by the dressing, the edge around the wound should be varnished with gum shellac and alcohol (one ounce to one pint), which gives it a water-proof finish. When the dressing becomes too slack it may be cut through te middle and tightened by a bandage, or removed entire, and a new dressing applied.

FRACTURES OF THE SHAFT OF THE FEMUR.

Perhaps in no other fractures has the plaster of paris proved more efficient than in these of the femur. A description of the method of application as practiced in Bellevue Hospital, and described by Samuel B. St. John, M. D.: A table must be obtained, and an upright bar about one inch in diameter secured to the middle of one end, so as to rise about two feet above the top of the table. In private practice I have used with entire satisfaction a wooden bar, one and a half inches in diameter, put through a hole bored in the end of a common kitchen table, and lashed to the braces below. This upright should be thinly padded, and covered evenly with a roller bandage. If the padding is too thick it will be in the way; if too thin, the bar may excoriate or cause undue pressure. The patient is to be placed upon the table with the nates well down towards the edge, and the upright projecting between the thighs, so that the perineum rests tightly against it. My practice has been to place another table in a line with the first, and abutting against it, to support the legs of the patient, and to afford a point from which to make extension. The provision for extension may be made in one of two ways—by traction upon a so-called Butt's extension apparatus, or adhesive plaster, which should be with a very strong but narrow loop from malleolus to malleolus; or, what is preferable, by a clove hitch around a stout plaster splint put on the day previous, from the toes to the knee. A blanket previously spread upon the table should then be cut, and drawn around the injured thigh so as to form a neat covering, and a strip eight inches wide should be extended around the pelvis; this should be drawn smooth, and stretched around the limb. A strip of blanket, one foot wide and four feet long (the relay) folded upon itself twice, so as to make a strip three inches wide, should be placed with its center resting against the perineum, and the ends brought obliquely upwards and outward, the interior passing over the crest of the illium, and the posterior over and above the tuberosity of the ischium. While the blanket is being adjusted, the patient should be etherized, and as soon as fully relaxed, extension should be made, the scrotum (in the case of a man) having been carefully drawn up and toward the sound side. The upright bar should be made to bear a little to one side of the median to avoid injury to the urethra, and especial care must be taken that the blanket strip, called the relay, should rest symmetrically in the flexure of the thigh and perineum, so as to present a groove along its center in which the upper edge of the inner side of the upright may rest. Extension is to be made by compound pulleys until the leg is by actual measurement as long as its fellow. If the lower part of the splint has been put on previously, as mentioned above, a trap may be cut over the malleolus to obtain a point from which to measure.

Before applying the extension strongly, however, the pelvis should be raised about four inches from the table by means of a stout sling passing around the upper part of the pelvis, and over a stout wooden bar stretched from the top of the upright to a stool about two and a half feet high, placed upon the table above the patient's head. Pillows should be placed under the head and shoulders to elevate them to a line with the pelvis. The bandages are now to be applied, piecing out the part already on, and completing the splint as you pass upward. Pains must be taken to make a neat splice, and not to allow the relay to become displaced. Small pieces of blanket soaked in plaster "cream" may often be used with advantage to strengthen the anterior and antero-lateral positions of the upper part. Felt or paste board may also be used for the same purpose, being worked in between the layers. About ten bandages in all will be sufficient, including those used in making the lower half. After the application is complete, the patient should be lowered to the table, but the extension should be kept up until the splint is quite hard. The projecting ends of the blanket should then be cut off, and the perineal edge trimmed with oil silk to prevent injury by wetting, especially by children.

Fractures of the radius should be dressed as follows: Extend the forearm and adjust the fragments, flex the hand towards the ulna, and apply first a dry roller from the top of the fingers to the elbow, then the plaster rollers in the same manner; three are usually sufficient. To prevent compressing the fragments towards the ulna, a firm pressure should be made on either side of the arm with thin strips of board, until the splint hardens. In fractures of both bones of the forearm, the dressing should be the same as above described, except the hand should be on a line with the forearm.

Fractures of the humerus should be dressed in the following manner: Cut a piece of blanket in the shape of a Physic splint, long enough to reach from the wrist to the top of the shoulder, and stitch firmly around the arm, cutting away the superfluous portion at the axilla; then apply the roller from the wrist to the axilla and over the shoulder. Three layers of bandage are sufficient. When the muscles are powerful, requiring greater extension than the dressing affords, a weight attached at the elbow, as advised by the late Professor Clark, of St. Louis, will serve the purpose well. In fractures of the ribs the plaster of paris has proved very efficient. The bandages should be applied very firmly, so as to prevent motion of the ribs during respiration.

Fractures of the clavicle may be treated with the plaster-of paris bandage, applied in the same manner as that recommended in the use of the adhesive plaster. In this connection I wish to record my experience in treating three cases of the fracture of the clavicle by simple method, which consists in tieing the hand of the affected side securely behind the patient, in this manner: Take a bandage four yards long, secure one end to the wrist and draw the hand behind the back, palm outwards, forearm obliquely upwards toward the sound shoulder; pass the bandage around the body and over the hand, where it should be stitched; from thence over the sound shoulder and down in front to the bandage, where it should be stitched and passed around the body again, and over the hand, stitching at each point where it crosses. For the first twenty-four hours the patient will complain of the confined hand, but after that there is comparative comfort. In all three cases (children under twelve years old) the bone united without any lapping or drooping of the shoulder.

It will be necessary to notice briefly at what time a fracture should be dressed with the immovable apparatus. Simple fractures, where there is no great amount of contusion of the soft parts, and no danger of sloughing, should be dressed at once; but in compound fractures, where there is danger of sloughing, a provisional dressing should be applied, and among the best, if not the best, is the old-fashioned fracture box and bran dressing, which should be replaced by the immovable dressing as soon as the limb is in condition to do so.

A few statistics will be appropriate as to the time of union. In 142 cases taken from the Bellevue Hospital records, 121 cases were treated at the beginning with immovable dressing, average time of union 38½ days; 68 cases treated with fracture box or other loose splint from one to two weeks, average time of union 47 days; and 50 cases of fracture of the femur, taken from the same records, the average shortening in all 50 cases was ⅛ inch—the average shortening before treatment 1¼ inch; 35 were patients who went about on crutches, average shortening ¼ inch. The average time of union in all 50 cases, to time of leaving off splint, 44 days.—*Monthly Journal Southern Illinois Medical Association.*

